# All-trans retinoic acid increases the pathogenicity of the H9N2 influenza virus in mice

**DOI:** 10.1186/s12985-022-01809-y

**Published:** 2022-06-28

**Authors:** Xiaofei Niu, Hongyan Wang, Lihong Zhao, Pengjing Lian, Yu Bai, Jingyun Li, Jian Qiao

**Affiliations:** 1 Department of Pathophysiology, College of Veterinary Medicine, China Agricultural University, No. 2 Yuanmingyuan West Road, Haidian District, Beijing, 100193 People’s Republic of China; 2 Department of Veterinary Medicine, College of Life Sciences and Food Engineering, Hebei University of Engineering, No. 19 Taiji Road, Economic and Technological Development Zone, Handan, 056038 People’s Republic of China

**Keywords:** ATRA, H9N2 virus, Pathogenicity, Mice

## Abstract

**Background:**

The H9N2 virus can infect not only birds but also humans. The pathogenicity of H9N2 virus infection is determined by an excessive immune response in the lung. All-trans retinoic acid (ATRA), the active metabolite of vitamin A, plays an important regulatory role and has been widely used in the clinical practice. This study was aimed to investigate whether ATRA could regulate the immune response to H9N2 virus infection in the lungs of mice, thereby reducing the pathogenicity of the H9N2 virus in mice.

**Methods:**

Mice were infected intranasally with H9N2 virus, and injected intraperitoneally with 0.2 mL of ATRA at low (1 mg/kg), medium (5 or 10 mg/kg), or high therapeutic dose (20 mg/kg), and toxic dose (40, 60, or 80 mg/kg), once per day for 10 days. Clinical signs, survival rates, and lung gross pathology were compared between the ATRA-treated H9N2-infected group, the ATRA group, and the H9N2-infected group, to investigate the effect of different doses of ATRA on the pathogenicity of H9N2 virus. Additionally, the viral load and cytokine concentration of lungs were measured at 3, 5, 7, and 9 days after infection, to investigate the potential mechanism of ATRA in affecting the pathogenicity of the H9N2 virus. Expression levels of cellular retinoic acid-binding protein 1 (CRABP1), cellular retinoic acid-binding protein 2 (CRABP2), and Retinoic acid-inducible gene-I (RIG-I) were detected using Western blotting.

**Results:**

The ATRA-treated H9N2-infected mice showed more severe clinical signs compared with the H9N2-infected group. The medium and high therapeutic doses of ATRA reduced the survival rates, aggravated lung tissue damage, decreased the expression of interferon beta (IFN-β), and increased the concentrations of interleukin-1 beta (IL-1β), tumor necrosis factor alpha (TNF-α), and C-C motif chemokine ligand 2 (CCL2) in the lungs of the H9N2-infected mice. At the same time, the expression patterns of CRABP1, CRABP2, and RIG-I were changed in mice infected by H9N2 and treated with different concentrations of ATRA.

**Conclusions:**

Our findings suggest that the therapeutic dose of ATRA can increase the pathogenicity of the H9N2 virus. Therefore, the consequences of those infected by influenza virus would be more severe after ATRA treatment.

**Supplementary Information:**

The online version contains supplementary material available at 10.1186/s12985-022-01809-y.

## Background

The H9N2 influenza virus, the most widespread subtype of influenza viruses in poultry, has been prevalent in China since 1994, and has transmitted widely around the world in multiple avian species [1]. Even though the H9N2 virus is a low-pathogenicity virus, it has been linked with high mortality due to co-infections with other pathogens [2]. It is noteworthy that the H9N2 virus has been occasionally isolated from pigs, mink, and humans with influenza-like illness in China since 1998 [3–8], indicating that the H9N2 virus was able to cross the species barrier, thereby expanding its host range from avian to mammalian. In addition, H9N2 virus’s six internal genes were found to facilitate the evolution of novel influenza viruses, such as H7N9, H10N8, and H5N6 viruses, and cause severe human respiratory infections [9–11]. Therefore, the H9N2 virus may be a potential human influenza pandemic candidate, posing a substantial threat to public health [12].

All-trans retinoic acid (ATRA), the biologically active metabolite of vitamin A, plays an important regulatory role in embryonic development, organ growth, metabolism, cell proliferation and differentiation, apoptosis, and immune response regulation [13–15]. ATRA has been widely used in clinical practice as a nutritional supplement and therapeutic agent, such as vitamin A supplementation for pregnant women and children [16–18], dry eye diseases [19], and skin diseases (psoriasis, dermatitis, etc.) [20]. In addition, ATRA is also applied for the prevention and treatment of different types of cancers [21, 22], particularly the acute promyelocytic leukemia (APL), which has achieved great success treated by ATRA combined with chemotherapeutic agents [23, 24].

ATRA plays its regulatory role via binding to and activating nuclear retinoic acid receptors (RARs) and retinoid X receptors (RXRs) [25], which are widely distributed in lung tissues. Based on this fact, ATRA regulates the growth, differentiation, and gene expression of lung epithelial cells, and is pivotal for alveolar development, the maintenance of lung alveoli throughout life, and alveolar regeneration in respiratory diseases [26]. In addition, ATRA also regulates the immune response in the lungs. As reported, ATRA exerts an anti-inflammatory effect on pulmonary parainfluenza virus infection, the underlying mechanism of which lies in reducing inflammation and improving alveolar epithelium regeneration in the lung of emphysematous rats [27]. Since the pathogenicity of influenza virus infection in mice is determined by the overreaction of the lung, it is necessary to explore whether ATRA can reduce excessive inflammation during influenza virus infection. The majority of previous studies have focused on the effect of ATRA deficiency on influenza virus infection, or the effect of ATRA on the immunity of influenza vaccines [28–33]. Yet, no study has reported on the susceptibility to influenza viruses of large groups of ATRA-treated people in clinical practice, and the importance of ATRA in the pathogenicity of influenza virus during influenza season remains obscure.

The aim of our study was to investigate the effects of different doses of ATRA on the pathogenicity of the H9N2 virus in mice, providing guidance for ATRA treatment on the prevention of influenza virus infection, which has important public health implications.

## Materials and methods

### Experimental design

To investigate whether ATRA affects the pathogenicity of the H9N2 virus in mice, two sets of experiments were done (Additional file 1: Fig. S1). Based on a simple randomization method, mice in both sets were further divided into the four groups as follows: group I—blank group which administered sterile phosphate-buffered saline (PBS) inoculation and cottonseed oil injection, group II—ATRA group which administered sterile PBS inoculation and ATRA injection, group III—H9N2-infected group which administered H9N2 virus inoculation and cottonseed oil injection, and group IV—ATRA-treated H9N2-infected group which administered H9N2 virus inoculation and ATRA injection.

To determine the effect of different doses of ATRA on the pathogenicity of the H9N2 virus, mice were inoculated intranasally with low-dose H9N2 virus [10^5^ median tissue culture infective dose (TCID_50_) in 100 µL per mouse] to simulate a natural infection of H9N2 virus, followed by intraperitoneally injection with ATRA at a low (1 mg/kg), medium (5 or 10 mg/kg), or high therapeutic dose (20 mg/kg), or a toxic dose (40, 60, or 80 mg/kg) at 0–9 days after inoculation (n = 18–22 per group). The mice were monitored for 19 days, and their body weight, food intake, survival rate, and lung gross pathology were compared between the groups.

To further differentiate the effects of low and medium therapeutic doses of ATRA on the pathogenicity of the H9N2 virus, mice were inoculated intranasally with medium-dose H9N2 virus (10^5.5^ TCID_50_ in 100 µL per mouse), followed by intraperitoneally injection of the low (1 mg/kg) or medium (5 or 10 mg/kg) therapeutic dose of ATRA at 0–9 days after inoculation. The mice (n = 22–24 per group) were monitored for 19 days, and their clinical signs (body weight, food intake), survival rate, pulmonary pathological changes (gross pathology, histopathology) were compared between the ATRA-treated H9N2-infected group, the ATRA group, and the H9N2-infected group. In addition, lung viral load and cytokine concentrations were measured at 3, 5, 7, and 9 days after H9N2 virus infection (n = 5 per group), to assess the ability of the lungs to clear the virus and the innate immunity level, so as to investigate the potential mechanism of ATRA in affecting the pathogenicity of the H9N2 virus in mice. In all experiments, the mice were treated in the same order each time.

### Mice

Specific pathogen-free (SPF) male BALB/c mice (6- to 8-week-old) were purchased from the Beijing Vital River Laboratory Animal Technology Company Limited (China). Mice were housed in microisolator cages ventilated under negative pressure with HEPA-filtered air. Water and food were offered ad libitum. All animal experiments were approved by the Laboratory Animal Welfare and Animal Experimental Ethical Committee of the China Agricultural University.

### ATRA administration

World Health Organization (WHO) recommended oral supplementation at 30–60 mg of ATRA for infant children 6–59 months of age [17]. The dosage of ATRA adjuvant chemotherapy for APL was 45 mg/m^2^ [23]. As reported, the median lethal dose (LD_50_) of ATRA in Swiss mice was 31 mg/kg by intraperitoneal injection [34], and the equivalent dose ratio for human and mice was 1:0.0026 based on the default weights of humans and mice (70 kg and 20 g) as well as their body surface areas. Considering the above facts along with the bioavailability of different administering routes, we determined that the intraperitoneal injection doses of ATRA were 1, 5, 10, 20, 40, 60, and 80 mg/kg. In addition, ATRA was divided into four doses according to its clinical application: low (1 mg/kg), medium (5 or 10 mg/kg), high (20 mg/kg), and toxic dose (40, 60, or 80 mg/kg). The ATRA powder (Sigma, St. Louis, MO, USA. R2625) was dissolved in dimethyl sulfoxide (DMSO), further diluted in cottonseed oil (Sigma, St. Louis, MO, USA. C7767) at the appropriate concentrations, and then stored at 4 °C. Mice were intraperitoneally injected with 0.2 mL ATRA solution once a day for 10 days, while the control group was injected with the same volume of cottonseed oil.

### Virus

The influenza A virus strain used in this study was A/Chicken/Hebei/4/2008 (H9N2) isolated by our laboratory [35, 36]. The complete genome sequences of the virus are available from GenBank under accession numbers FJ499463-FJ499470. The viruses were propagated in 9-day SPF embryonated eggs at 37 °C for 72 h, and the allantoic fluid was collected, centrifuged, and stored at -80 °C for further use. The virus titers were determined by TCID_50_ on Madin-Darby canine kidney (MDCK) cells, and calculated by the method of Reed and Muench, as described previously [37, 38].

### Virus infection

A low dose of H9N2 virus, which was close to that in natural infection, was adopted to investigate the effect of different doses of ATRA on the pathogenicity of H9N2 virus, observe the interaction between different doses of ATRA and the H9N2 virus, and simulate the infection under natural conditions. In addition, to further differentiate the effects of different therapeutic doses of ATRA on the pathogenicity of the H9N2 virus, a medium dose of H9N2 virus was used. For infection experiments, the H9N2 virus was diluted into different concentrations with sterile PBS, and inoculated intranasally in a volume of 100 µL into mice that were lightly anesthetized with isoflurane. In this study, 10^5^ TCID_50_ was used as the low-dose H9N2 virus, which would not cause mouse death but significant clinical symptoms, and 10^5.5^ TCID_50_ was used as the medium-dose H9N2 virus, which could cause death in half of the mice (that is LD_50_).

### Viral load measurement

Mice were euthanized by isoflurane overdose, and then the whole lungs were removed, weighed, and homogenized in 1.3 mL sterile PBS. The homogenates were centrifuged, filtered through a 0.22 µm cellulose acetate membrane, serial tenfold dilutions made, and then titrated on MDCK cells. Viral loads were reported as the TCID_50_/mL of lung homogenate. Finally, lung viral load was measured at 3, 5, 7, and 9 days after H9N2 virus infection.

### Cytokine analysis in lung

The lung homogenates collected at 3, 5, 7, and 9 days after H9N2 virus infection were centrifuged at 4 °C and 5000 rpm for 15 min, and the supernatants were stored at − 80 °C. Concentrations of TNF-α, IL-1β, interferon alpha (IFN-α), IFN-β, CCL2 and C-X-C motif chemokine ligand 10 (CXCL10) were measured using mouse Quantikine ELISA Kits (R&D systems, Minneapolis, MN, USA).

### Western blot assay

The expression of CRABP1, CRABP2 and RIG-I at protein level was detected by western blotting. As described above, the lung homogenates collected at 3, 5, 7 and 9 days after H9N2 virus infection were lysed with RIPA lysis buffer (Cell Signaling Technology, Danvers, USA), quantity of total proteins were measured by BCA kit. The lysates were centrifuged at 14,000*g*, and the supernatants were separated by SDS-PAGE and transferred onto polyvinylidene difluoride (PVDF) membranes. Then the membranes were blocked with 3% bovine albumin and probed with monoclonals antibody to β-actin (Wuhan Sanying, Wuhan, China), CRABP1 (Wuhan Sanying, Wuhan, China), CRABP2 (Wuhan Sanying, Wuhan, China) and RIG-I (Abcam, Shanghai, China), followed by incubation with peroxidase conjugated secondary antibody (ORIGENE, Rockville, MD, USA). Proteins were visualized using enhanced chemiluminescence reagent (Cell Signaling Technology, Danvers, USA). The relative protein levels of CRABP1, CRABP2 and RIG-I to β-actin were performed by Image J software.

### Pulmonary histopathology

Mice were euthanized by overdose of isoflurane 5 days after infection. The lungs were removed, fixed in 10% neutral buffered formalin, and embedded in paraffin. The 5-µm tissue sections were deparaffinized, rehydrated, and stained with hematoxylin and eosin (H & E) [27].

### Statistical analysis

Statistical analyses were performed with the IBM SPSS Statistics v20, and graphics were performed by means of GraphPad Prism software v8.0. Data were presented as mean ± standard deviation (SD). One-way analysis of variance (ANOVA) with Bonferroni correction for multiple comparions and the log-rank test for survival rate were conducted. *P* values < 0.05 were considered statistically significant.

## Results

### Effects of H9N2 virus on mice

**Fig. 1:**
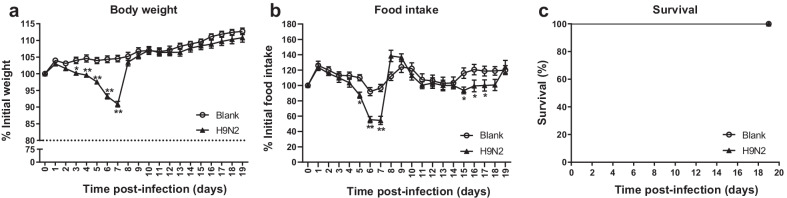
Effects of the H9N2 virus on body weight (**a**), food intake (**b**), and survival (**c**) of mice. Body weight is presented as mean of the rate of weight change (%) ± SD. Rate of weight change (%) = daily weight/initial weight × 100%. n = 10–11. Food intake is presented as mean of the change rate of food intake (%) ± SD. Change rate of food intake (%) = food intake per day/initial food intake × 100%. n = 10–11. Survival rate (%) = the number of mice alive/total number of mice observed. n = 20. Mice that lost more than 30% of their original body weight were euthanized and considered dead on that day. *P < 0.05, **P < 0.01, analyzed with one-way ANOVA or the log-rank test. Blank, mice receiving sterile PBS inoculation and cottonseed oil injection at 0–9 days after inoculation; H9N2, mice receiving H9N2 virus (10^5^ TCID_50_) inoculation and cottonseed oil injection at 0–9 days after inoculation. ATRA, all-trans retinoic acid; TCID_50_, median tissue culture infective dose

The effects of the H9N2 virus on clinical signs and survival in mice were shown in Fig. 1 and Tables 1, 2 and 3. On day 3 after infection, some mice showed slight inactivity and ruffled fur. By day 5, most mice presented more severe inactivity, ruffled fur, inappetence, as well as signs of labored breathing and respiratory distress. Compared with the blank group, the body weight and food intake in H9N2-infected mice were significantly reduced by days 5–7 (*P* < 0.05), decreased to the lowest on day 7 (a decrease of 13.7% and 42.3%, respectively), and then quickly returned to normal thereafter. No mice died. Taken together, mice infected with the H9N2 virus showed mild and transient clinical symptoms.

**Table 1:** Effects of different doses of ATRA and H9N2 virus on the body weight of mice

Clinical application of ATRA	Groups	Dosage of ATRA (mg/kg)	Time post-infection (days)							
			3	4	5	6	7	8	9	19
	Blank^a^	0	104.0 ± 2.9	104.6 ± 3.3	104.0 ± 2.9	104.4 ± 3.1	104.6 ± 3.1	105.2 ± 3.9	106.7 ± 3.3	112.7 ± 3.1
Low therapeutic dose	ATRA1^b^	1	103.9 ± 1.1	104.9 ± 1.1	105.4 ± 1.3	104.6 ± 1.4	104.7 ± 1.8	105.7 ± 1.9	107.6 ± 2.5	112.7 ± 2.3
Medium therapeutic dose	ATRA2^c^	5	104.7 ± 1.9	105.2 ± 1.9	105.7 ± 2.1	104.6 ± 2.2	104.9 ± 1.8	106.0 ± 1.9	108.2 ± 2.4	111.1 ± 2.8
	ATRA3^d^	10	104.4 ± 1.7	104.7 ± 2.0	104.6 ± 2.1	104.3 ± 2.6	104.7 ± 1.8	105.5 ± 2.0	105.8 ± 2.1	108.9 ± 2.6
High therapeutic dose	ATRA4^e^	20	100.8 ± 3.2	100.3 ± 2.8^∆^	100.2 ± 2.7^∆^	98.8 ± 2.6^∆∆^	99.0 ± 3.1^∆∆^	99.3 ± 3.8^∆∆^	101.3 ± 3.1^∆∆^	105.4 ± 2.2^∆^
Toxic dose	ATRA5^f^	40	97.6 ± 2.7^∆∆^	93.9 ± 3.2^∆∆^	90.0 ± 3.6^∆∆^	85.1 ± 4.3^∆∆^	81.6 ± 4.0^∆∆^	76.5 ± 4.9^∆∆^	76.3 ± 4.8^∆∆^	—
	ATRA6^g^	60	96.1 ± 1.5^∆∆^	90.6 ± 2.1^∆∆^	87.5 ± 3.0^∆∆^	83.2 ± 2.9^∆∆^	79.5 ± 2.7^∆∆^	74.7 ± 2.6^∆∆^	73.9 ± 3.3^∆∆^	—
	ATRA7^h^	80	92.5 ± 2.5^∆∆^	87.1 ± 2.3^∆∆^	82.1 ± 2.5^∆∆^	76.5 ± 3.0^∆∆^	73.4 ± 2.8^∆∆^	—	—	—
	H9N2^i^	0	100.2 ± 1.2^∆^	99.6 ± 1.3^∆∆^	97.5 ± 1.2^∆∆^	93.2 ± 2.5^∆∆^	90.9 ± 2.8^∆∆^	103.5 ± 3.7	105.4 ± 3.1	110.8 ± 4.5
Low therapeutic dose	ATRA1 + H9N2^j^	1	98.8 ± 5.7	95.6 ± 7.7^#^	93.2 ± 7.6^##^	91.0 ± 9.5^##^	95.9 ± 12.3	100.2 ± 13.7	102.6 ± 10.9	109.9 ± 5.1
Medium therapeutic dose	ATRA2 + H9N2^k^	5	94.7 ± 2.9^##^**	91.5 ± 3.1^##^**	90.6 ± 5.6^##^*	93.0 ± 8.8^#^	94.3 ± 11.0	97.3 ± 12.4	101.6 ± 12.0	113.9 ± 5.4
	ATRA3 + H9N2^l^	10	93.5 ± 2.3^##^**	91.8 ± 3.1^##^**	92.2 ± 4.1^##^*	92.6 ± 6.2^##^	95.0 ± 7.0^#^	97.5 ± 7.4^#^	100.3 ± 7.6	109.5 ± 5.3
High therapeutic dose	ATRA4 + H9N2^m^	20	91.9 ± 4.1^##^**	90.4 ± 4.1^##^**	89.5 ± 3.8^##^**	90.2 ± 4.3^##^	92.3 ± 4.2^##^	92.9 ± 4.1^##^**	95.3 ± 4.2^##^**	103.3 ± 3.4**
Toxic dose	ATRA5 + H9N2^n^	40	89.2 ± 6.0^##^**	88.8 ± 7.1**	88.4 ± 7.3*	85.1 ± 6.6**	85.2 ± 4.0**	82.0 ± 4.6**	79.0 ± 3.6**	—
	ATRA6 + H9N2^o^	60	94.1 ± 2.3**	90.1 ± 3.4**	86.9 ± 3.5**	81.6 ± 2.9**	77.5 ± 4.3**	73.3 ± 2.9**	—	—
	ATRA7 + H9N2^p^	80	90.5 ± 3.3**	86.1 ± 2.8**	81.2 ± 3.0**	75.6 ± 3.2**	70.7 ± 0.8**	—	—	—

Data are presented as mean of the rate of weight change (%) ± SD. Rate of weight change (%) = daily weight/initial weight × 100%. n = 10–11. Statistical analyses were performed with one-way ANOVA and Bonferroni correction. ATRA group or H9N2 group compared with blank group: ∆, *P* < 0.05; ∆∆, *P* < 0.01; ATRA + H9N2 group compared with ATRA group: ^#^*P* < 0.05; ^##^*P* < 0.01; ATRA + H9N2 group compared with H9N2 group: *P < 0.05; **P < 0.01. a: Blank, mice receiving sterile PBS inoculation and cottonseed oil injection; b–h: ATRA1-ATRA7, mice receiving sterile PBS inoculation and ATRA injection (1, 5, 10, 20, 40, 60, and 80 mg/kg, respectively); i: H9N2, mice receiving H9N2 virus (10^5^ TCID_50_) inoculation and cottonseed oil injection; j–p: ATRA1 + H9N2-ATRA7 + H9N2, mice receiving H9N2 virus (10^5^ TCID_50_) inoculation and ATRA injection (1, 5, 10, 20, 40, 60, and 80 mg/kg, respectively). ATRA, all-trans retinoic acid; TCID_50_, median tissue culture infective dose

**Table 2:** Effects of different doses of ATRA and H9N2 virus on food intake in mice

Clinical application of ATRA	Groups	Dosage of ATRA (mg/kg)	Time post-infection (days)							
			3	4	5	6	7	8	9	19
	Blank^a^	0	113.1 ± 4.8	112.7 ± 4.7	110.0 ± 4.0	92.0 ± 5.2	96.7 ± 4.7	112.0 ± 5.6	124.0 ± 7.2	120.0 ± 5.9
Low therapeutic dose	ATRA1^b^	1	110.7 ± 5.9	109.5 ± 6.3	105.1 ± 6.1	87.4 ± 7.3	89.7 ± 7.6	98.8 ± 9.6	119.0 ± 4.4	122.8 ± 5.2
Medium therapeutic dose	ATRA2^c^	5	117.6 ± 6.3	114.3 ± 5.5	105.3 ± 6.1	91.8 ± 5.1	88.2 ± 5.6	100.4 ± 7.2	122.4 ± 8.6	119.9 ± 6.9
	ATRA3^d^	10	115.1 ± 5.6	112.3 ± 6.2	102.3 ± 9.1	90.5 ± 10.7	86.7 ± 8.9	100.4 ± 8.9	107.2 ± 7.9	109.9 ± 7.9
High therapeutic dose	ATRA4^e^	20	86.1 ± 9.4^∆∆^	80.3 ± 13.3^∆^	79.4 ± 13.8	65.3 ± 16.9	61.1 ± 16.2^∆∆^	63.9 ± 15.3^∆∆^	78.3 ± 12.2^∆∆^	87.9 ± 7.9^∆∆^
Toxic dose	ATRA5^f^	40	68.3 ± 6.0^∆∆^	43.0 ± 10.9^∆∆^	35.1 ± 12.4^∆∆^	17.7 ± 7.4^∆∆^	15.9 ± 7.6^∆∆^	15.9 ± 6.9^∆∆^	22.1 ± 7.8^∆∆^	—
	ATRA6^g^	60	54.6 ± 6.9^∆∆^	31.2 ± 7.3^∆∆^	24.1 ± 7.4^∆∆^	25.5 ± 7.0^∆∆^	18.1 ± 5.5^∆∆^	13.4 ± 5.0^∆∆^	19.5 ± 5.7^∆∆^	—
	ATRA7^h^	80	39.3 ± 7.1^∆∆^	13.2 ± 4.5^∆∆^	17.6 ± 7.1^∆∆^	6.9 ± 3.7^∆∆^	6.9 ± 3.7^∆∆^	—	—	—
	H9N2^i^	0	109.8 ± 5.9	103.6 ± 5.8	87.3 ± 4.0^∆^	55.4 ± 4.1^∆∆^	54.4 ± 5.3^∆∆^	138.4 ± 7.4	136.5 ± 4.8	124.1 ± 8.6
Low therapeutic dose	ATRA1 + H9N2^j^	1	87.5 ± 12.4^#^	63.9 ± 7.7^##^**	44.5 ± 11.8^##^**	79.8 ± 14.2	86.7 ± 14.8*	104.2 ± 14.9*	127.0 ± 12.4	133.7 ± 7.2
Medium therapeutic dose	ATRA2 + H9N2^k^	5	32.0 ± 5.3^##^**	36.8 ± 7.2^##^**	52.2 ± 7.2^##^**	78.6 ± 10.6*	86.8 ± 8.5**	109.5 ± 5.8**	136.1 ± 10.2	161.1 ± 11.1
	ATRA3 + H9N2^l^	10	32.4 ± 5.2^##^**	56.6 ± 7.4^##^**	67.9 ± 9.5^##^	79.1 ± 10.2*	100.5 ± 7.8**	117.7 ± 3.8*	118.6 ± 2.6*	134.0 ± 5.0^#^
High therapeutic dose	ATRA4 + H9N2^m^	20	46.4 ± 6.7^##^**	43.4 ± 11.2^##^**	36.6 ± 8.4^##^**	43.6 ± 8.3	59.6 ± 9.2	74.5 ± 9.8**	96.8 ± 6.1**	97.7 ± 6.4*
Toxic dose	ATRA5 + H9N2^n^	40	34.9 ± 7.6^##^**	39.0 ± 8.3**	54.6 ± 6.2**	41.8 ± 8.0^#^	43.4 ± 7.8^##^	44.4 ± 7.6^##^**	46.1 ± 7.4^#^**	—
	ATRA6 + H9N2^o^	60	24.5 ± 7.5^##^**	17.9 ± 6.5**	22.6 ± 7.4**	15.7 ± 5.8**	22.6 ± 6.6**	18.2 ± 5.9**	—	—
	ATRA7 + H9N2^p^	80	21.2 ± 5.7^#^**	9.1 ± 3.9**	6.6 ± 2.9**	4.8 ± 2.5**	3.0 ± 1.9**	—	—	—

Data are presented as mean of the change rate of food intake (%) ± SD. Change rate of food intake (%) = food intake per day/initial food intake × 100%. n = 10–11. Statistical analyses were performed with one-way ANOVA and Bonferroni correction. ATRA group or H9N2 group compared with blank group: ∆, *P* < 0.05; ∆∆, *P* < 0.01; ATRA + H9N2 group compared with ATRA group: ^#^*P* < 0.05; ^##^*P* < 0.01; ATRA + H9N2 group compared with H9N2 group: *P < 0.05; **P < 0.01. a: Blank, mice administered sterile PBS inoculation and cottonseed oil injection; b-h: ATRA1-ATRA7, mice administered sterile PBS inoculation and ATRA injection (1, 5, 10, 20, 40, 60, and 80 mg/kg, respectively); i: H9N2, mice administered H9N2 virus (10^5^ TCID_50_) inoculation and cottonseed oil injection; j-p: ATRA1 + H9N2-ATRA7 + H9N2, mice administered H9N2 virus (10^5^ TCID_50_) inoculation and ATRA injection (1, 5, 10, 20, 40, 60, and 80 mg/kg, respectively). ATRA, all-trans retinoic acid; TCID_50_, median tissue culture infective dose

**Table 3:** Effects of different doses of ATRA and H9N2 virus on the survival of mice

Clinical application of ATRA	Groups	Dosage of ATRA (mg/kg)	Time post-infection (days)							
			3	4	5	6	7	8	9	19
	Blank^a^	0	100 (20/20)	100 (20/20)	100 (20/20)	100 (20/20)	100 (20/20)	100 (20/20)	100 (20/20)	100 (20/20)
Low therapeutic dose	ATRA1^b^	1	100 (22/22)	100 (22/22)	100 (22/22)	100 (22/22)	100 (22/22)	100 (22/22)	100 (22/22)	100 (22/22)
Medium therapeutic dose	ATRA2^c^	5	100 (20/20)	100 (20/20)	100 (20/20)	100 (20/20)	100 (20/20)	100 (20/20)	100 (20/20)	100 (20/20)
	ATRA3^d^	10	100 (22/22)	100 (22/22)	100 (22/22)	100 (22/22)	100 (22/22)	100 (22/22)	100 (22/22)	100 (22/22)
High therapeutic dose	ATRA4^e^	20	100 (20/20)	100 (20/20)	100 (20/20)	100 (20/20)	100 (20/20)	100 (20/20)	100 (20/20)	100 (20/20)
Toxic dose	ATRA5^f^	40	100 (18/18)	100 (18/18)	100 (18/18)	100 (18/18)	100 (18/18)	89 (16/18)	56 (10/18)	0 (0/18)^∆∆^
	ATRA6^g^	60	100 (22/22)	100 (22/22)	100 (22/22)	100 (22/22)	100 (22/22)	82 (18/22)	27 (6/22)	0 (0/22)^∆∆^
	ATRA7^h^	80	100 (22/22)	100 (22/22)	100 (22/22)	100 (22/22)	27 (6/22)	0 (0/22)	0 (0/22)	0 (0/22)^∆∆^
	H9N2^i^	0	100 (20/20)	100 (20/20)	100 (20/20)	100 (20/20)	100 (20/20)	100 (20/20)	100 (20/20)	100 (20/20)
Low therapeutic dose	ATRA1 + H9N2^j^	1	100 (22/22)	100 (22/22)	100 (22/22)	100 (22/22)	100 (22/22)	100 (22/22)	100 (22/22)	100 (22/22)
Medium therapeutic dose	ATRA2 + H9N2^k^	5	100 (22/22)	100 (22/22)	100 (22/22)	95 (21/22)	91 (20/22)	91 (20/22)	91 (20/22)	91 (20/22)
	ATRA3 + H9N2^l^	10	100 (22/22)	100 (22/22)	95 (21/22)	91 (20/22)	91 (20/22)	91 (20/22)	91 (20/22)	91 (20/22)
High therapeutic dose	ATRA4 + H9N2^m^	20	100 (22/22)	100 (22/22)	95 (21/22)	95 (21/22)	91 (20/22)	91 (20/22)	91 (20/22)	91 (20/22)
Toxic dose	ATRA5 + H9N2^n^	40	100 (22/22)	91 (20/22)	91 (20/22)	91 (20/22)	82 (18/22)	82 (18/22)	73 (16/22)	0 (0/22) ^##^**
	ATRA6 + H9N2^o^	60	100 (22/22)	100 (22/22)	100 (22/22)	100 (22/22)	100 (22/22)	45 (10/22)	0 (0/22)	0 (0/22) ^##^**
	ATRA7 + H9N2^p^	80	100 (22/22)	100 (22/22)	100 (22/22)	91 (20/22)	36 (8/22)	0 (0/22)	0 (0/22)	0 (0/22)**

Data are presented as survival rate (%) (the number of mice alive/total number of mice observed). n = 18–22. Statistical analyses were performed with the log-rank test. ATRA group or H9N2 group compared with blank group: ∆, *P* < 0.05; ∆∆, *P* < 0.01; ATRA + H9N2 group compared with ATRA group: ^#^*P* < 0.05; ^##^*P* < 0.01; ATRA + H9N2 group compared with H9N2 group: *P < 0.05; **P < 0.01. a: Blank, mice administered sterile PBS inoculation and cottonseed oil injection; b–h: ATRA1-ATRA7, mice administered sterile PBS inoculation and ATRA injection (1, 5, 10, 20, 40, 60, and 80 mg/kg, respectively); i: H9N2, mice administered H9N2 virus (10^5^TCID_50_) inoculation and cottonseed oil injection; j–p: ATRA1 + H9N2-ATRA7 + H9N2, mice administered H9N2 virus (10^5^ TCID_50_) inoculation and ATRA injection (1, 5, 10, 20, 40, 60, and 80 mg/kg, respectively). Mice that lost more than 30% of their original body weight were euthanized and considered dead on that day. ATRA, all-trans retinoic acid; TCID_50_, median tissue culture infective dose

### Effects of different doses of ATRA on mice

**Fig. 2:**
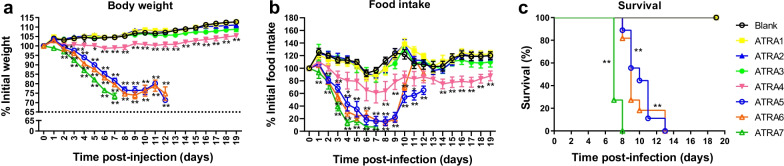
Effects of different doses of ATRA on body weight (**a**), food intake (**b**), and survival (**c**) of mice. Body weight is presented as mean of the rate of weight change (%) ± SD. Rate of weight change (%) = daily weight/initial weight × 100%. n = 10–11. Food intake is presented as mean of the change rate of food intake (%) ± SD. Change rate of food intake (%) = food intake per day/initial food intake × 100%. n = 10–11. Survival rate (%) = the number of mice alive/total number of mice observed. n = 18–22. Mice that lost more than 30% of their original body weight were euthanized and considered dead on that day. *P < 0.05; **P < 0.01, analyzed with one-way ANOVA or the log-rank test. Blank, mice receiving sterile PBS inoculation and cottonseed oil injection at 0–9 days after inoculation; ATRA1-ATRA7, mice receiving sterile PBS inoculation and ATRA injection (1, 5, 10, 20, 40, 60, and 80 mg/kg, respectively) at 0–9 days after inoculation. ATRA, all-trans retinoic acid; TCID_50_, median tissue culture infective dose

The effects of different doses of ATRA on mouse clinical signs and survival were shown in Fig. 2 and Tables 1, 2 and 3. Compared with the blank group, the ATRA-treated groups (1, 5, and 10 mg/kg) showed no obvious clinical signs and no death (*P* > 0.05). The ATRA-treated group (20 mg/kg) had ruffled fur on day 4 after the injection, with reduced activity, loss of appetite, and gradually wasting away. Their body weight and food intake were significantly reduced compared with the blank group (*P* < 0.05). On day 7, the food intake dropped to the minimum level (decreased by 35.6%). No mice died. The ATRA-treated groups (40, 60, and 80 mg/kg) presented severe clinical signs, including dry skin, rapid wasting, decreased activity, and loss of appetite from 2–3 days after injection. Their body weight and food intake continued to decrease until death. Their body weight and survival were significantly different from those of the blank group (*P* < 0.01). Taken together, mice showed obvious clinical symptoms when injected with high therapeutic dose of ATRA (20 mg/kg), and died when injected with a toxic dose of ATRA (more than 40 mg/kg).

### Effects of different doses of ATRA and H9N2 virus on mice

#### Effects of different doses of ATRA and H9N2 virus on clinical signs and survival of mice

Compared with the H9N2-infected group without ATRA administration, the body weight of the 1 mg/kg ATRA-treated H9N2-infected group was not significantly different (*P* > 0.05, Table 1), and the food intake decreased significantly on days 4–5 (*P* < 0.01) and days 7–8 (*P* < 0.05), with the lowest on day 5 (decreased by 42.8%) after infection (Table 2). The body weight of the 5, and 10 mg/kg ATRA-treated H9N2-infected groups was significantly different on days 3–5 after infection (*P* < 0.05), and decreased to the lowest on day 5 (decreased by 6.9%) and day 4 (decreased by 7.8%), respectively (Table 1). Food intake decreased significantly on day 3 after infection (decreased by 77.8 and 77.4%, Table 2). Compared with the 1, 5, and 10 mg/kg ATRA groups, the body weight and food intake of the 1, 5, and 10 mg/kg ATRA-treated H9N2-infected groups decreased significantly on days 3–5 (*P* < 0.05), by 12.2–15.1% and 34.4–60.6% on day 5 after infection, respectively (Tables 1, 2). There were no significant differences between the 1, 5, and 10 mg/kg ATRA-treated H9N2-infected groups and the H9N2-infected group, or the 1, 5, and 10 mg/kg ATRA groups in terms of survival rate (*P* > 0.05, Table 3).

Compared with the H9N2-infected group, the body weight and food intake of the 20 mg/kg ATRA-treated H9N2-infected group significantly decreased by 8.0% and 50.7% on day 5 after infection, respectively (*P* < 0.01); however, there was no significant difference in survival rate (*P* > 0.05, Tables 1, 2, 3). Compared with the 20 mg/kg ATRA group, the body weight and food intake of the 20 mg/kg ATRA-treated H9N2-infected group were significant decreased on days 3–5 after infection by 10.7% and 42.8% on day 5 (*P* < 0.01, Tables 1, 2); however, there was no significant difference found in survival rate (*P* > 0.05, Table 3).

The body weight, food intake, and survival rate of the 40, 60, and 80 mg/kg ATRA-treated H9N2-infected groups continued to decline, and finally all mice died. Compared with the H9N2-infected group, the body weight of the 40, 60, and 80 mg/kg ATRA-treated H9N2-infected groups significantly decreased by 9.1%, 10.6% and 16.3%, on day 5 after infection, respectively (*P* < 0.05, Table 1). The food intake of the 40, 60, and 80 mg/kg ATRA-treated H9N2-infected groups significantly decreased by 32.7%, 64.7%, and 80.7% on day 5 after infection, respectively (*P* < 0.01, Table 2). There were significant differences between the 40, 60, and 80 mg/kg ATRA-treated H9N2-infected groups and the H9N2-infected group without ATRA administration. Compared with the 40, 60, and 80 mg/kg ATRA groups, the body weight of the 40, 60, and 80 mg/kg ATRA-treated H9N2-infected groups decreased by 8.4% (*P* < 0.01), 2.0% (*P* > 0.05), and 2.0% (*P* > 0.05), respectively; the food intake decreased by 33.4% (*P* < 0.01), 30.1% (*P* < 0.01), and 18.1% (*P* < 0.05), respectively, on day 3 after infection, and the survival rates were significantly different (*P* < 0.01), except between the 80 mg/kg ATRA group and the 80 mg/kg ATRA-treated H9N2-infected group (Tables 1, 2, 3).

Taken together, both the therapeutic and toxic doses of ATRA injection could increase the pathogenicity of the H9N2 virus in mice. With the increase of the toxic doses of ATRA, there was no significant difference between the ATRA-treated H9N2-infected group and the ATRA group.

#### Effects of different doses of ATRA and H9N2 virus on gross pathology of lungs of mice

**Fig. 3:**
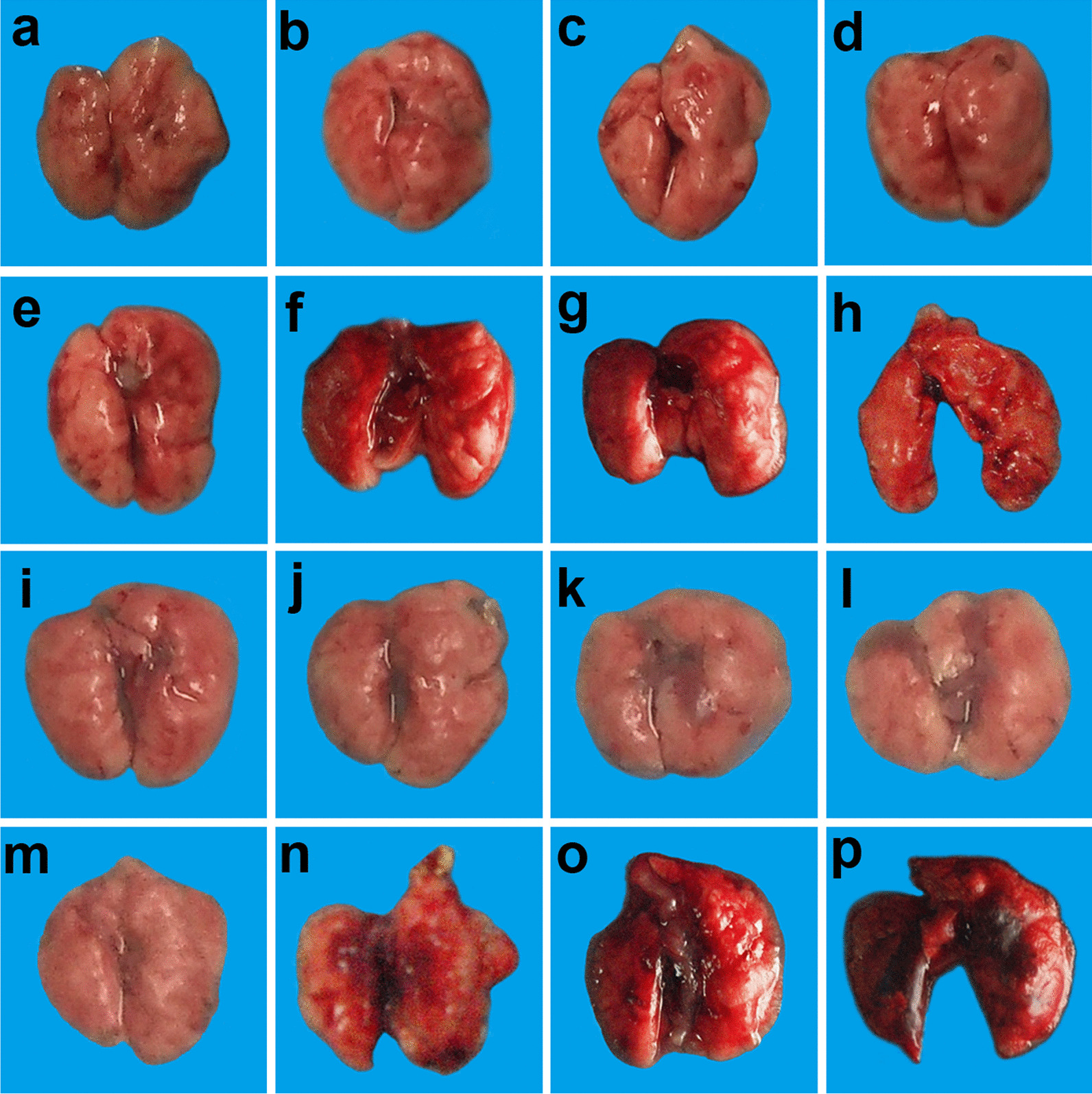
Effects of different doses of ATRA and H9N2 virus on the gross pathology of lungs of mice. **f**–**h** and **n**–**p** are the lungs of dead mice, and the rest are the lungs of mice at 19 days after inoculation. n = 3–5. **a** Blank group, mice receiving sterile PBS inoculation and cottonseed oil injection at 0–9 days after inoculation; **b**–**h** ATRA1-ATRA7 group, mice receiving sterile PBS inoculation and ATRA injection (1, 5, 10, 20, 40, 60, and 80 mg/kg, respectively) at 0–9 days after inoculation; **i** H9N2 group, mice receiving H9N2 virus (10^5^ TCID_50_) inoculation and cottonseed oil injection at 0–9 days after inoculation; **j**–**p** ATRA1 + H9N2-ATRA7 + H9N2 group, mice receiving H9N2 virus (10^5^ TCID_50_) inoculation and ATRA injection (1, 5, 10, 20, 40, 60, and 80 mg/kg, respectively) at 0–9 days after inoculation. ATRA, all-trans retinoic acid; TCID_50_, median tissue culture infective dose

The gross pathology of lung in mice was observed on the 19^th^ day after infection. The results showed that the 1, 5, and 10 mg/kg ATRA groups showed no significant changes in the lungs compared with the blank group (Fig. 3a–d). The 20 mg/kg ATRA group had mild damage to the lungs (Fig. 3e). The mice in the 40, 60, and 80 mg/kg ATRA groups that died had pulmonary hyperemia (Fig. 3f–h).

The lungs of the H9N2-infected group showed only a small amount of fibrosis on day 19 compared to the blank group (Fig. 3a, i).

The lungs of the 1 mg/kg ATRA-treated H9N2-infected group showed no significant change compared with those of the H9N2-infected group (Fig. 3i, j). The 5 and 10 mg/kg ATRA-treated H9N2-infected groups showed severe pulmonary fibrosis (Fig. 3k, l). The 20 mg/kg ATRA-treated H9N2-infected group had no significant changes compared with those in the H9N2-infected group (Fig. 3m). The mice in the 40, 60, and 80 mg/kg ATRA-treated H9N2-infected groups that died presented severe pulmonary congestion (Fig. 3n–p).

Taken together, the medium therapeutic and toxic dose of ATRA injection could aggravate pulmonary damages caused by the H9N2 virus to mice.

### Effects of therapeutic doses of ATRA on the pathogenicity of H9N2 virus to mice

#### Effects of therapeutic doses of ATRA on clinical signs and survival of mice infected with H9N2 virus

**Fig. 4:**
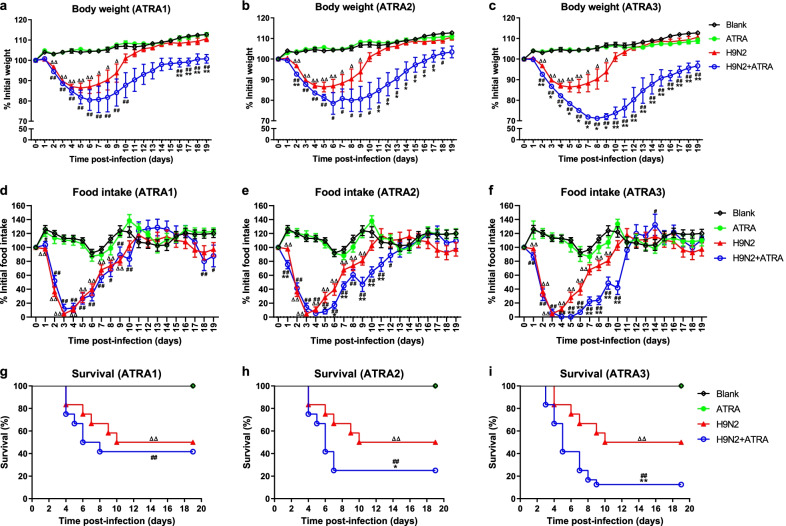
Effects of therapeutic dose of ATRA on clinical signs and survival of mice infected with H9N2 virus. (**a**–**c**) Body weight is presented as mean of the rate of weight change (%) ± SD. Rate of weight change (%) = daily weight/initial weight × 100%. n = 10–12. (**d**–**f**) Food intake is presented as mean of the change rate of food intake (%). Change rate of food intake (%) = food intake per day/initial food intake × 100%. n = 10–12. (**g**–**i**) Survival rate (%) = the number of mice alive/total number of mice observed. n = 22–24. Mice that lost more than 30% of their original body weight were euthanized and considered dead on that day. Data were analyzed with one-way ANOVA or the log-rank test. ATRA group or H9N2 group compared with blank group: ∆, *P* < 0.05; ∆∆, *P* < 0.01; ATRA + H9N2 group compared with ATRA group: ^#^*P* < 0.05; ^##^*P* < 0.01; ATRA + H9N2 group compared with H9N2 group: *P < 0.05; **P < 0.01. Blank, mice receiving sterile PBS inoculation and cottonseed oil injection at 0–9 days after inoculation; ATRA1-ATRA3, mice receiving sterile PBS inoculation and ATRA injection (1, 5, and 10 mg/kg, respectively) at 0–9 days after inoculation; H9N2, mice receiving H9N2 virus (10^5.5^ TCID_50_) inoculation and cottonseed oil injection at 0–9 days after inoculation; ATRA1 + H9N2-ATRA3 + H9N2, mice receiving H9N2 virus (10^5.5^ TCID_50_) inoculation and ATRA injection (1, 5, and 10 mg/kg, respectively) at 0–9 days after inoculation. ATRA, all-trans retinoic acid; TCID_50_, median tissue culture infective dose

To further differentiate the effect of low (1 mg/kg) and medium (5 and 10 mg/kg) therapeutic doses of ATRA on the clinical signs and survival rate of H9N2 virus, mice were inoculated with 10^5.5^ TCID_50_ H9N2 virus, followed by the injection with 1, 5, and 10 mg/kg ATRA, respectively. The results showed that there were no significant changes in body weight, food intake, or survival rate in the 1, 5, and 10 mg/kg ATRA groups compared with the blank group (*P* > 0.05, Fig. 4). However, after infected with the H9N2 virus, their body weight, food intake, and survival rate gradually decreased. Compared with the blank group, the body weight of ATRA-treated mice was significantly different at 2–9 days after H9N2 infection (*P* < 0.05), and the survival rate was 50%, which was significant different from the blank group (*P* < 0.01, Fig. 4).

The 1 mg/kg ATRA-treated H9N2-infected group showed a slight decrease in body weight compared with the H9N2-infected group, and the difference was significant on days 16–19 after H9N2 infection (*P* < 0.01). Compared with the 1 mg/kg ATRA group, their body weight was significantly different at 2–10 days and 16–19 days after H9N2 infection (*P* < 0.01, Fig. 4a). The survival rate of the 1 mg/kg ATRA-treated H9N2-infected group was 41.7%, which showed no significant difference with the H9N2-infected group (*P* > 0.05), but a significant difference with the 1 mg/kg ATRA group (*P* < 0.01, Fig. 4g).

The body weight of the 5, and 10 mg/kg ATRA-treated H9N2-infected groups decreased significantly compared with the H9N2-infected group (*P* < 0.05). Compared with the 5, and 10 mg/kg ATRA groups, the body weight of the ATRA-treated H9N2-infected groups also significantly decreased (*P* < 0.05, Fig. 4b, c). The survival rate of the 5, and 10 mg/kg ATRA-treated H9N2-infected groups (25% and 12.5%, respectively) significantly decreased compared with the H9N2-infected group and the 5, and 10 mg/kg ATRA groups (*P* < 0.05, *P* < 0.01, *P* < 0.01, respectively, Fig. 4h, i).

Thus, low and medium therapeutic doses of ATRA could aggravate clinical signs in the H9N2-infected mice. In addition, a medium therapeutic dose of ATRA could reduce the survival rate of the H9N2-infected mice.

#### Effects of therapeutic dose of ATRA on lung gross pathology and histopathology of mice infected with H9N2 virus

**Fig. 5:**
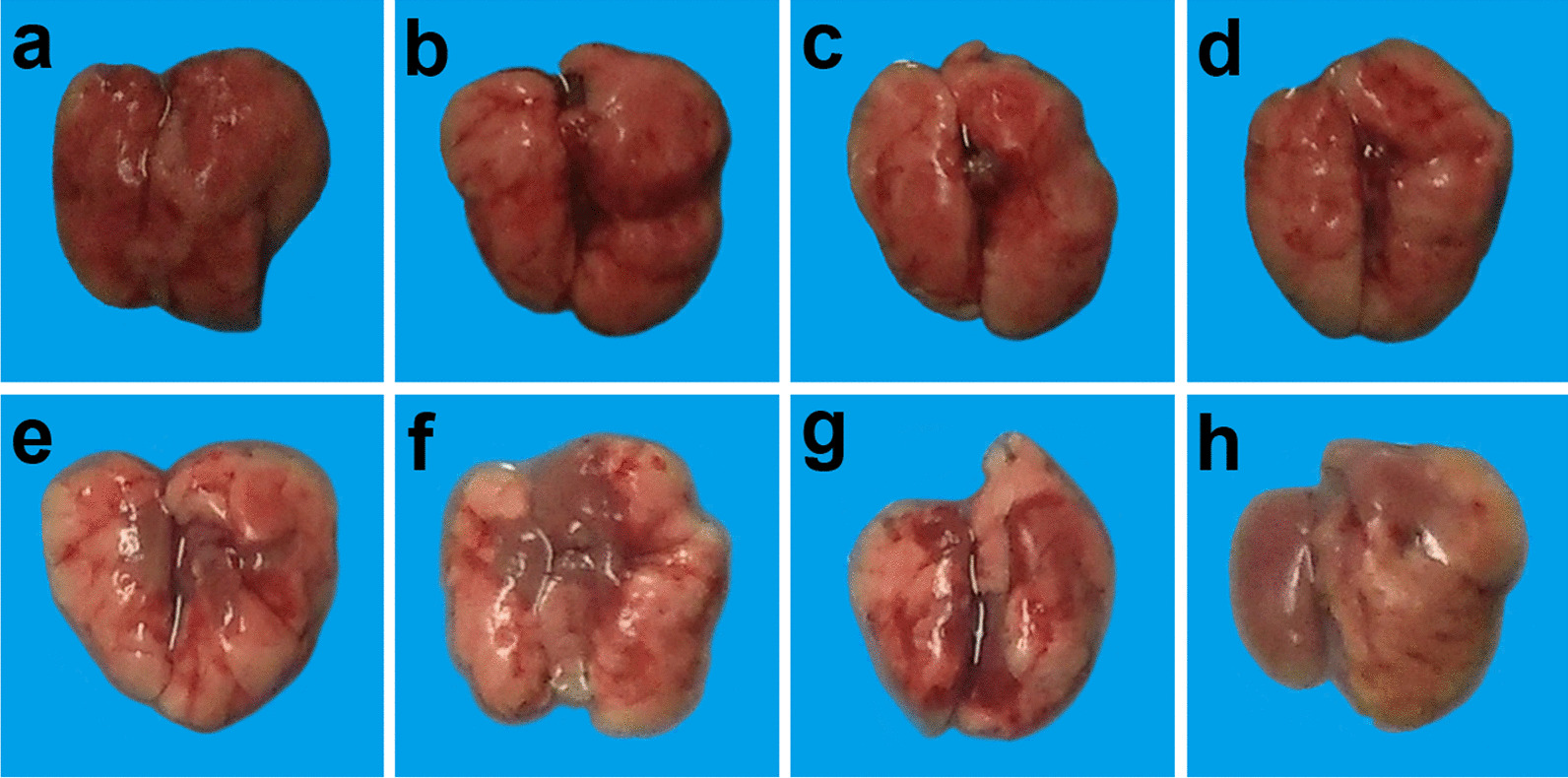
Effects of therapeutic dose of ATRA on lung gross pathology of mice infected with H9N2 virus. **a**–**h** are the lungs of mice at 19 days after inoculation. **a** Blank group, mice receiving sterile PBS inoculation and cottonseed oil injection at 0–9 days after inoculation; **b**–**d** ATRA1-ATRA3 group, mice receiving sterile PBS inoculation and ATRA injection (1, 5, and 10 mg/kg, respectively) at 0–9 days after inoculation; **e**. H9N2 group, mice receiving H9N2 virus (10^5.5^ TCID_50_) inoculation and cottonseed oil injection at 0–9 days after inoculation; **f**–**h**. ATRA1 + H9N2-ATRA3 + H9N2 group, mice receiving H9N2 virus (10^5.5^ TCID_50_) inoculation and ATRA injection (1, 5, and 10 mg/kg, respectively) at 0–9 days after inoculation. n = 5. ATRA, all-trans retinoic acid; TCID_50_, median tissue culture infective dose

**Fig. 6:**
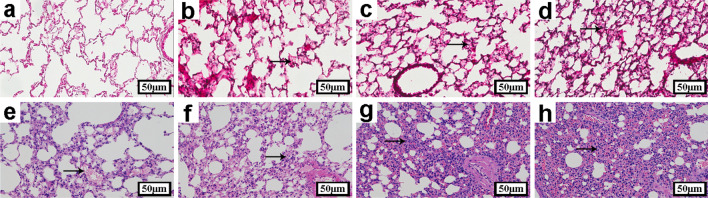
Effects of therapeutic dose of ATRA on lung histopathology of mice infected with H9N2 virus. **a**–**h** are histopathology images of the lungs of mice at 5 days after inoculation. a. Blank group, mice receiving sterile PBS inoculation and cottonseed oil injection at 0–9 days after inoculation; **b**–**d** ATRA1-ATRA3 group, mice receiving sterile PBS inoculation and ATRA injection (1, 5, and 10 mg/kg, respectively) at 0–9 days after inoculation; **e** H9N2 group, mice receiving H9N2 virus (10^5.5^ TCID_50_) inoculation and cottonseed oil injection at 0–9 days after inoculation; **f**–**h** ATRA1 + H9N2-ATRA3 + H9N2 group, mice receiving H9N2 virus (10^5.5^ TCID_50_) inoculation and ATRA injection (1, 5, and 10 mg/kg, respectively) at 0–9 days after inoculation. H&E stain. Original magnification × 200. n = 5. ATRA, all-trans retinoic acid; TCID_50_, median tissue culture infective dose

The gross pathology (Fig. 5) and histopathology (Fig. 6) of lungs showed that there were no significant changes in the lungs of the 1, and 5 mg/kg ATRA-treated groups compared with the blank group (Figs. 5a–c, 6a–c). The lungs of the 10 mg/kg ATRA-treated group had mild damage in the lungs, which was apparent in the slight thickening of the alveolar diaphragm on day 5 (Figs. 5d, 6d).

The lungs of the H9N2-infected group showed hyperemia and infiltration of inflammatory cells on day 5, and a small amount of fibrosis on day 19 (Figs. 5e, 6e).

Compared with the H9N2-infected group, with the increase of the doses of ATRA, the degree of lesions in pulmonary tissues in the 1, 5, and 10 mg/kg ATRA-treated H9N2-infected groups gradually increased and characterized by pulmonary congestion and hemorrhage, enhanced inflammation, thickened alveolar diaphragmatic, and severe damage to alveolar structure on day 5 (Fig. 6e–h); besides, the severity of pulmonary fibrosis increased by day 19 (Fig. 5e–h).

Therefore, the low and medium therapeutic doses of ATRA could aggravate lung tissues’ damage in the H9N2-infected mice.

#### Effects of therapeutic dose of ATRA on viral load of mice infected with H9N2 virus

**Fig. 7:**
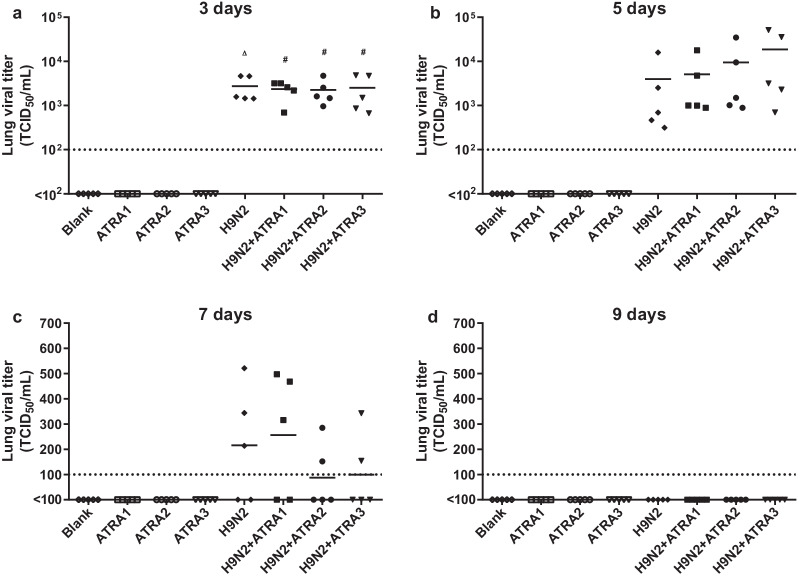
Effects of therapeutic dose of ATRA on viral load of mice infected with H9N2 virus. The viral load of mice injected with ATRA1, ATRA2, and ATRA3 was test at 3 (**a**), 5 (**b**), 7 (**c**), and 9 (**d**) days after H9N2 virus (10^5.5^ TCID_50_) infection, respectively. Data were analyzed with one-way ANOVA, and presented as mean. ATRA group or H9N2 group compared with blank group: ∆, *P* < 0.05; ∆∆, *P* < 0.01; ATRA + H9N2 group compared with ATRA group: ^#^*P* < 0.05; ^##^*P* < 0.01; ATRA + H9N2 group compared with H9N2 group: *P < 0.05; **P < 0.01. n = 5. Blank, mice receiving sterile PBS inoculation and cottonseed oil injection at 0–9 days after inoculation; ATRA1-ATRA3, mice receiving sterile PBS inoculation and ATRA injection (1, 5, and 10 mg/kg, respectively) at 0–9 days after inoculation; H9N2, mice receiving H9N2 virus (10^5.5^ TCID_50_) inoculation and cottonseed oil injection at 0–9 days after inoculation; ATRA1 + H9N2-ATRA3 + H9N2, mice receiving H9N2 virus (10^5.5^ TCID_50_) inoculation and ATRA injection (1, 5, 10 mg/kg, respectively) at 0–9 days after inoculation. ATRA, all-trans retinoic acid; TCID_50_, median tissue culture infective dose

The lung viral loads of the 1, 5, and 10 mg/kg ATRA-treated H9N2-infected groups were slightly higher than that of the H9N2-infected group at 3, 5, and 7 days after infection, but the difference was not significant (*P* > 0.05). Compared with the 1, 5, and 10 mg/kg ATRA-treated groups, the lung viral loads of the 1, 5, and 10 mg/kg ATRA-treated H9N2-infected groups significantly increased 3 days after H9N2 infection (*P* < 0.05, Fig. 7), suggesting that the low and medium therapeutic doses of ATRA did not affect the ability of H9N2-infected mice to clear pathogens from the lungs.

#### Effects of therapeutic dose of ATRA on cytokine concentrations of mice infected with H9N2 virus

**Fig. 8:**
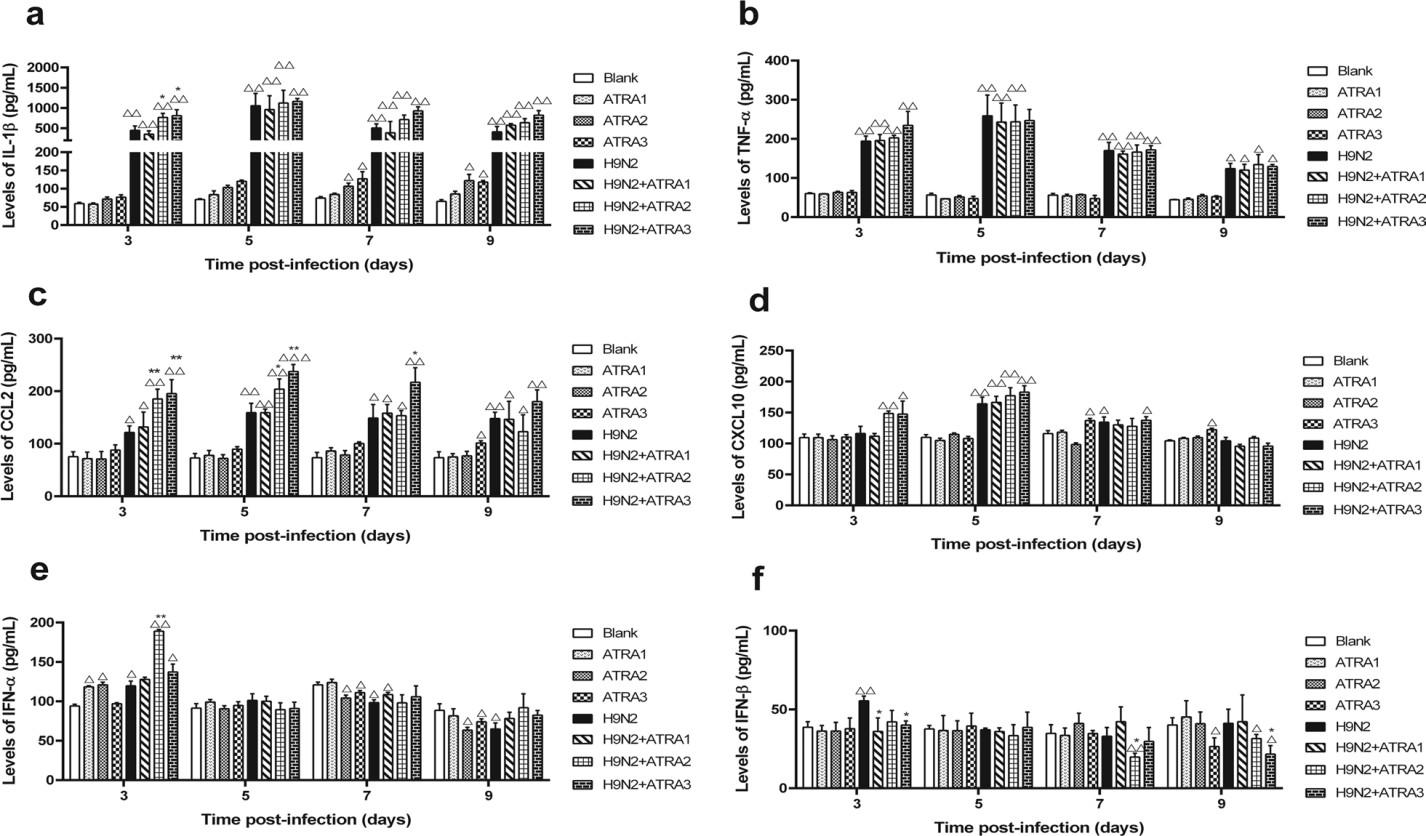
Effects of a therapeutic dose of ATRA on cytokine concentrations in mice infected with the H9N2 virus. Expression levels of IL-1β (**a**), TNF-α (**b**), CCL2 (**c**), CXCL10 (**d**), IFN-α (**e**), and IFN-β (**f**) were test by ELISA kits at 3, 5, 7, and 9 days after H9N2 virus (10^5.5^ TCID_50_) infection. Data were analyzed with one-way ANOVA, and presented as mean ± SD. Compared with blank group: ∆, *P* < 0.05; ∆∆, *P* < 0.01; Compared with H9N2 group: *P < 0.05; **P < 0.01. n = 5. Blank, mice receiving sterile PBS inoculation and cottonseed oil injection at 0–9 days after inoculation; ATRA1-ATRA3, mice receiving sterile PBS inoculation and ATRA injection (1, 5, and 10 mg/kg, respectively) at 0–9 days after inoculation; H9N2, mice receiving H9N2 virus (10^5.5^ TCID_50_) inoculation and cottonseed oil injection at 0–9 days after inoculation; ATRA1 + H9N2-ATRA3 + H9N2, mice receiving H9N2 virus (10^5.5^ TCID_50_) inoculation and ATRA injection (1, 5, and 10 mg/kg, respectively) at 0–9 days after inoculation. ATRA, all-trans retinoic acid; TCID_50_, median tissue culture infective dose; IL-1β, interleukin-1 beta; TNF-α, tumor necrosis factor alpha; CCL2, C-C motif chemokine ligand 2; CXCL10, C-X-C motif chemokine ligand 10; IFN-α, interferon alpha; IFN-β, interferon beta

Compared with the blank group, the levels of IL-1β, TNF-α, and CCL2 in the lung tissue of the H9N2-infected mice were significantly increased at 3, 5, 7 and 9 days (*P* < 0.05) (Fig. 8a–c), and the levels of CXCL10 were significantly increased at 5 and 7 days (*P* < 0.05) (Fig. 8d), whereas the levels of IFN-α and IFN-β were only significantly increased at 3 days post-infection (*P* < 0.05) (Fig. 8e, f). After treated with 1 mg/kg ATRA, the mice only showed a significant increase in IFN-α at 3 days in lung tissues (*P* < 0.05) (Fig. 8e), without any significant change in the expressions of other cytokines. After the mice were treated with 5 mg/kg ATRA, the expression of IL-1β was significantly increased at 7 and 9 days (*P* < 0.05) (Fig. 8a), and the levels of IFN-α were significantly increased at 3 days (*P* < 0.05), but decreased at 7 and 9 days (*P* < 0.05) (Fig. 8e). After the mice were treated with 10 mg/kg ATRA, the levels of IL-1β, and CXCL10 were significantly increased at 7 and 9 days (*P* < 0.05) (Fig. 8a, d), the level of CCL2 was significantly increased at 9 days (*P* < 0.05) (Fig. 8c), the levels of IFN-α were significantly reduced at 7 and 9 days (*P* < 0.05) (Fig. 8e), and the IFN-β level was significantly reduced at 9 days (*P* < 0.05) (Fig. 8f). Compared to H9N2-infected group, in the 1 mg/kg ATRA-treated H9N2-infected group, only IFN-β was significantly decreased at 3 days in lung tissue (*P* < 0.05) (Fig. 8f), and there were no significant changes in IL-1β, TNF-α, CCL2, CXCL10, and IFN-α at 3, 5, 7, and 9 days (*P* > 0.05) (Fig. 8a–e). In the 5 mg/kg ATRA-treated H9N2-infected group, the IL-1β level was significantly increased at 3 days (*P* < 0.05) (Fig. 8a), the levels of CCL2 were significantly increased at 3 and 5 days (*P* < 0.05) (Fig. 8c), and IFN-β was significantly decreased at 7 days (*P* < 0.05) (Fig. 8f). In the 10 mg/kg ATRA-treated H9N2-infected group, the expression of IL-1β was significantly increased on day 3 (*P* < 0.05) (Fig. 8a), the levels of CCL2 were significantly increased at 3, 5, and 7 days (*P* < 0.05) (Fig. 8c), and the levels of IFN-β were significantly decreased at 3 and 9 days (*P* < 0.05) (Fig. 8f).

Thus, the medium and high therapeutic dose of ATRA could significantly improve the pulmonary inflammatory response of the H9N2-infected mice.

#### Effects of therapeutic dose of ATRA on CRABP1, CRABP2, and RIG-I in mice infected with H9N2 virus

**Fig. 9:**
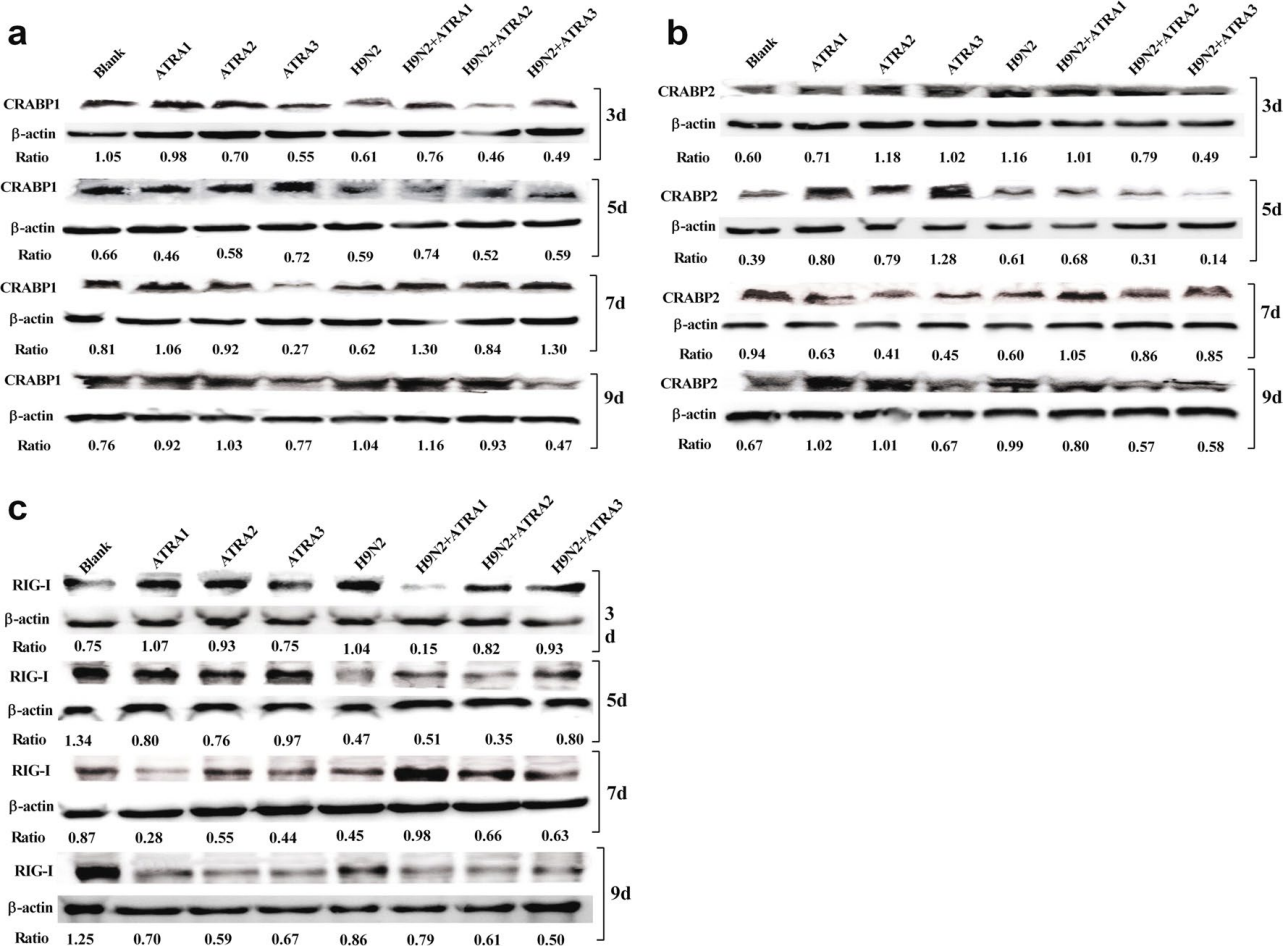
Effects of a therapeutic dose of ATRA on CRABP1, CRABP2, and RIG-I expression in mice infected with the H9N2 virus. Mice were treated with ATRA, H9N2 virus, or H9N2 virus and ATRA. Expression levels of CRABP1, CRABP2, and RIG-I were tested by Western blotting at 3, 5, 7, and 9 days. **a** Expression of CRABP1 at protein level in mice. **b** Expression of CRABP2 at protein level in mice. **c** Expression of RIG-I at protein level in mice. Blank, mice receiving sterile PBS inoculation and cottonseed oil injection at 0–9 days after inoculation; ATRA1-ATRA3, mice receiving sterile PBS inoculation and ATRA injection (1, 5, and 10 mg/kg, respectively) at 0–9 days after inoculation; H9N2, mice receiving H9N2 virus (10^5.5^ TCID_50_) inoculation and cottonseed oil injection at 0–9 days after inoculation; ATRA1 + H9N2-ATRA3 + H9N2, mice receiving H9N2 virus (10^5.5^ TCID_50_) inoculation and ATRA injection (1, 5, and 10 mg/kg, respectively) at 0–9 days after inoculation. ATRA, all-trans retinoic acid; TCID_50_, median tissue culture infective dose; CRABP1, cellular retinoic acid-binding protein 1; CRABP2, cellular retinoic acid-binding protein 2; RIG-I, retinoic acid-inducible gene-I

As shown in Fig. 9, compared with the blank group, the expression level of CRABP1 in mice induced by H9N2 infection decreased at 3 and 5 days, while the expression level of CRABP2 increased at the same time. The expression level of RIG-I increased at day 3 and decreased at days 5, 7, and 9 (Fig. 9c). After the mice were treated with 1 or 5 mg/kg ATRA, the expression level of CRABP1 decreased at 3 and 5 days, increasing at 7 and 9 days (Fig. 9a). Compared with the H9N2-infected group, the expression level of CRABP1 was increased at 3, 5, 7, and 9 days (Fig. 9a) in the 1 mg/kg ATRA-treated H9N2-infected group. The expression levels of CRABP1, CRABP2, and RIG-I decreased at 3, 5 and 9 days, but increased at 7 days in the 5 mg/kg ATRA-treated H9N2-infected group (Fig. 9a–c).

## Discussion

In the present study, we investigated whether ATRA could reduce the pathogenicity of the H9N2 virus, by modulating the pulmonary immune response. In the first part, mice were infected with low-dose H9N2 virus to simulate a natural infection of H9N2 virus, followed by the administration of different doses of ATRA. Firstly, we evaluated the clinical manifestations of mice infected with H9N2 virus alone or mice given ATRA alone. We found that compared with the blank group, the body weight and food intake of the H9N2-infected group were significantly reduced on days 5–7, and then quickly returned to normal. Compared with the blank group, the 1, 5, and 10 mg/kg ATRA-treated groups showed no obvious clinical symptoms, the 20 mg/kg ATRA-treated group showed significant body weight and food intake declines, and the 40, 60, and 80 mg/kg ATRA-treated groups showed severe poisoning symptoms, eventually resulting in the death, which were consistent with the results of a previous study by Hixson EJ et al. reporting that the LD_50_ was 31 mg/kg in Swiss mice after intraperitoneal injection of ATRA for 21 days [34]. Secondly, we evaluated the clinical manifestations of mice infected with the H9N2 virus and further administered ATRA. Interestingly, we found that the ATRA-treated H9N2-infected group showed more severe clinical signs (including reduced body weight and food intake, and severe lung injury) compared with the H9N2-infected group. Apparently, contrary to our expectations, ATRA increased rather than decreased the pathogenicity of H9N2 in mice.

In the second part, we further investigated the effect of the low (1 mg/kg) and medium (5, and 10 mg/kg) therapeutic doses of ATRA on the pathogenicity of increasing doses of H9N2 virus. We found that the low and medium therapeutic doses of ATRA could aggravate the clinical signs and damages to lung tissues in H9N2-infected mice; furthermore, the medium therapeutic dose of ATRA could reduce their survival rate. These results are consistent with the results of our first experiment. Influenza virus infection is characterized by the recruitment of large numbers of innate immune cells to the lungs that release large numbers of proinflammatory cytokines and chemokines [39]. To investigate the potential mechanism of ATRA in affecting the pathogenicity of the H9N2 virus in mice and to assess the ability of the lungs to clear the virus as well as the innate immunity level, the viral load and cytokine concentrations in the lung were measured after the mice were infected with H9N2 virus. We found that the low and medium therapeutic doses of ATRA had no effect on viral loads in H9N2-infected mice, but significantly increased the concentration of IL-1β. The innate immune response triggered by the influenza virus results in the production of pro-inflammatory cytokines, which could cause local and systemic inflammation and exert a protective effect by inducing and regulating the adaptive immune response[40, 41]. However, an exaggerated innate immune response characterized by a “cytokine storm” due to excessive production of pro-inflammatory cytokines may lead to immunopathological damages, acute respiratory distress syndrome (ARDS), and a high mortality rate [42]. TNF-α and IL-1β, two potent pro-inflammatory cytokines [43], play an important role in the early stage of the inflammatory response to the infection of influenza virus [44–48]. Chemokines are involved in attracting leukocytes from the circulatory system to migrate to inflamed tissues [49]. The present study found that the expression levels of chemokines significantly increased in response to the infection of H9N2 virus. Besides, the medium and high therapeutic doses of ATRA further increased the levels of chemokines, especially CCL2. A study found that mice with deletions in chemokine genes or their receptors had reduced pulmonary damages and improved survival when infected by influenza strains [50]. In the present study, the survival rate of mice co-treated with ATRA and H9N2 virus was significantly reduced, possibly due to accelerated chemokine production. The interferon system has been recognized as the body’s first important defense system against viral infections. Previous studies have shown that influenza virus could induce interferon’s production after respiratory epithelial cells and giant cells were invaded by it [51]. As shown in Fig. 8e, when the mice were treated with 1, or 5 mg/kg ATRA for 3 days, the levels of IFN-α and IFN-β were significantly increased, while no significant changes were found in those treated with 10 mg/kg ATRA, suggesting that the effect may be related to the dose level of ATRA. Similarly, the expression levels of IFN-α and IFN-β were significantly increased at 3 days after H9N2 virus infection, which is believed to be the body’s normal innate immune response. However, the inflammatory response induced by H9N2 infection suppressed the normal innate immune response over time, resulting in progressively lower expression levels of IFN-α and IFN-β in mice co-treated with H9N2 virus-infection and ATRA. We speculated that the presence of inflammatory factors and chemokines might lead to changes in the internal environment of lung tissues, which ultimately affects the regulatory effect of ATRA on immune function. Based on the above facts, ATRA did not reduce the excessive inflammation caused by the H9N2 virus in the lung, but increased it.

CRABP1, CRABP2, and RIG-I have been reported to be all involved in the process of ATRA regulation of the immune response in mammals. ATRA up-regulates the expression of RIG-I in a RARα-dependent manner, and enhances the signaling cascade mediated by RIG-I, which induces expression of type I IFN and proinflammatory cytokines [52, 53]. CRABP1 mediates the degradation of ATRA by enzymes of the CYP26 family of enzymes in the cytochrome P450 enzyme superfamily, and CRABP2 mediates ATRA nuclear entry and binding to the nuclear receptor RAR [54]. Our results indicated that the expression of CRABP1 was decreased at 3 days after H9N2 virus infection, however, the opposite was found in the expression of CRABP2, suggesting that the infection of H9N2 virus could affect the intracellular content of ATRA and the downstream pathways by inhibiting its degradation and promoting its nuclear entry. Consistent with the trend for IFN-α, the expression level of RIG-I increased at 3 days but decreased at 5, 7, and 9 days after the infection of H9N2 virus, which is believed to be related with the innate immune response initiated by H9N2 infection. After the mice were infected by H9N2 virus and treated with 1 mg/kg ATRA, the expression of CRABP1, and CRABP2 increased at 3, 5, and 7 days. We speculated that this maintained the intracellular homeostasis of ATRA, so that it does not exacerbate the symptoms of H9N2 infection. However, after the mice were infected by H9N2 virus and treated with 5 or 10 mg/kg ATRA, the expression of CRABP1, CRABP2, and RIG-I decreased, which may exacerbate the inflammatory response by affecting the content of ATRA and the RIG-I pathway. Of course, the increased pro-inflammatory cytokines in the ATRA and H9N2-infected co-treatment groups may also result from other signaling pathways being affected.

At present, the research on ATRA antiviral effect mainly focuses on (-) ssRNA virus, including measles virus, mumps virus, canine distemper virus and Newcastle disease virus [55, 56]. The main mechanism of ATRA’s antiviral effect is to increase the expression of type I interferon by activating the RIG-I signaling pathway, thereby enhancing the innate immune response to inhibit virus replication [57]. However, unlike the above viruses, H9N2 avian influenza virus is one kind of immunosuppressive virus that can induce immune response imbalance and immune organ damage by inducing an inflammatory factor response, or inhibit the function of T cells by inducing the expression of programmed death-ligand 1 (PD-L1) [58, 59]. This may be one of the reasons why ATRA could not enhance the resistance to H9N2 virus infection in mice.

Taken together, our findings suggested an inconsistent result with previous studies on the role of ATRA in pulmonary inflammation. The increased pathogenicity of the H9N2 virus caused by ATRA may be attributed to H9N2 infection and the further treatment of ATRA, which affected the expression patterns of CRABP1, CRABP2, and RIG-I, and in turn resulted in an aggravated immunopathological damage to the lung.

## Conclusions

The therapeutic dose of ATRA have a potential in increasing the pathogenicity of the H9N2 virus in mice, which might be the underlying reason for the more severe consequences of ATRA treatment for those infected with the influenza virus.

## Supplementary Information

**Additional file 1:** Flow chart of the experiment.

## Data Availability

All data generated or analysed during this study are included in this published article.

## Abbreviations

ATRA: All-trans retinoic acid
CCL2: C-C motif chemokine ligand 2
CXCL10: C-X-C motif chemokine ligand 10
CRABP1: Cellular retinoic acid-binding protein 1
CRABP2: Cellular retinoic acid-binding protein 2
IFN-α: Interferon alpha
IFN-β: Interferon beta
IL-1β: Interleukin-1 beta
LD_50_: Median lethal dose
PBS: Phosphate-buffered saline
RIG-I: Retinoic acid-inducible gene-I
TCID_50_: Median tissue culture infective dose
TNF-α: Tumor necrosis factor alpha
