# Supplementary figures and images for: All-trans retinoic acid increases the pathogenicity of the H9N2 influenza virus in mice
Source: Virol J. 2022 Jun 28;19:113. doi: 10.1186/s12985-022-01809-y (PMC9238145; doi:10.1186/s12985-022-01809-y)

## Flow chart

**a**

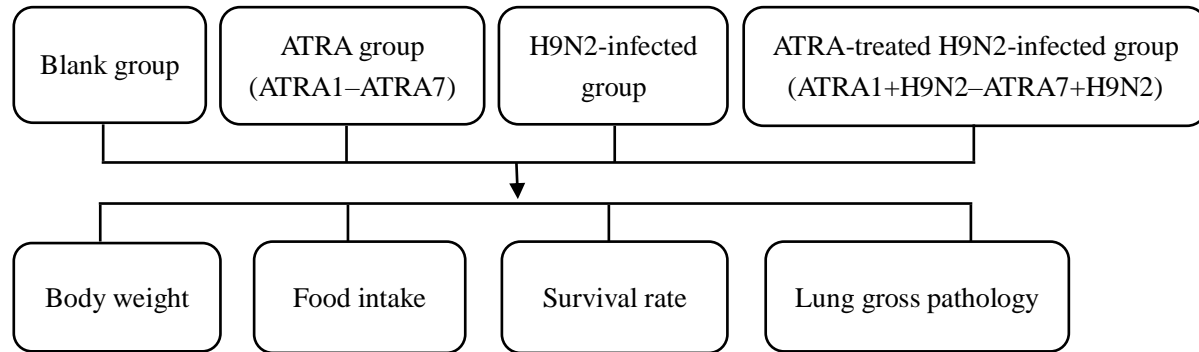

**b**

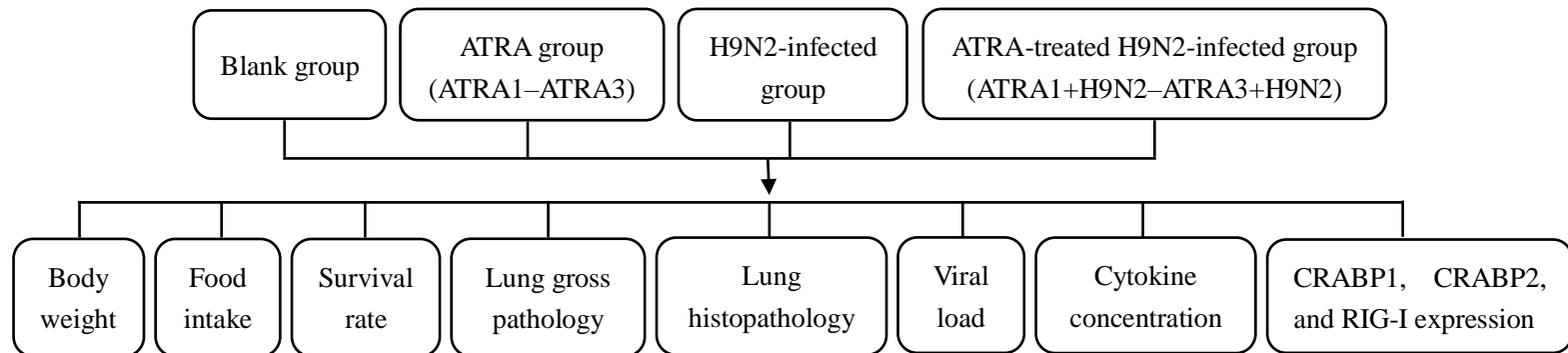

Supplement: Supplementary file 1 — Additional file 1: Flow chart of the experiment. [file 12985_2022_1809_MOESM1_ESM.pdf]
